# Recent Advances in Biopolymeric Composite Materials for Tissue Engineering and Regenerative Medicines: A Review

**DOI:** 10.3390/molecules26030619

**Published:** 2021-01-25

**Authors:** Muhammad Umar Aslam Khan, Saiful Izwan Abd Razak, Wafa Shamsan Al Arjan, Samina Nazir, T. Joseph Sahaya Anand, Hassan Mehboob, Rashid Amin

**Affiliations:** 1 Department of Polymer Engineering and Technology, University of the Punjab, Lahore 54590, Punjab, Pakistan; 2 School of Biomedical Engineering and Health Sciences, Faculty of Engineering, Universiti Teknologi Malaysia, Skudai 81300, Johor, Malaysia; saifulizwan@utm.my; 3 School of Biomedical Engineering, Med-X Research Institute, Shanghai Jiao Tong University (SJTU), 1954 Huashan Road, Shanghai 200030, China; 4 Centre for Advanced Composite Materials, Universiti Teknologi Malaysia, Skudai 81300, Johor, Malaysia; 5 Department of Chemistry, College of Science, King Faisal University, P.O. Box 400, Al-Ahsa 31982, Saudi Arabia; walarjan@kfu.edu.sa (W.S.A.A.); seegasami.samina@gmail.com (S.N.); 6 Sustainable and Responsive Manufacturing Group, Faculty of Mechanical and Manufacturing Engineering Technology, Universiti Teknikal Malaysia Melaka, Hang Tuah Jaya, Melaka 76100, Malacca, Malaysia; anand@utem.edu.my; 7 Department of Engineering Management, College of Engineering, Prince Sultan University, Rafha Street, P.O. Box 66833, Riyadh 11586, Saudi Arabia; hmehboob@psu.edu.sa; 8 Department of Biology, College of Sciences, University of Hafr Al Batin, Hafar Al-Batin 39524, Saudi Arabia

**Keywords:** biopolymers, composite materials, tissue engineering, regenerative materials, wound dressing

## Abstract

The polymeric composite material with desirable features can be gained by selecting suitable biopolymers with selected additives to get polymer-filler interaction. Several parameters can be modified according to the design requirements, such as chemical structure, degradation kinetics, and biopolymer composites’ mechanical properties. The interfacial interactions between the biopolymer and the nanofiller have substantial control over biopolymer composites’ mechanical characteristics. This review focuses on different applications of biopolymeric composites in controlled drug release, tissue engineering, and wound healing with considerable properties. The biopolymeric composite materials are required with advanced and multifunctional properties in the biomedical field and regenerative medicines with a complete analysis of routine biomaterials with enhanced biomedical engineering characteristics. Several studies in the literature on tissue engineering, drug delivery, and wound dressing have been mentioned. These results need to be reviewed for possible development and analysis, which makes an essential study.

## 1. Introduction

Recently, biopolymers have attained enormous attention with perspective multifunctional and high-performance biocomposites having a low environmental impact with unique properties like, abundantly available, renewable, eco-friendly, and lightweight. Biopolymeric composites should substitute synthetic materials in optics, biochemistry, and biomedical engineering with versatile applications, and investment and research on these materials increase significantly. Biopolymers and biodegradable synthetic polymers have attracted researchers’ enormous attention in recent years [1,2]. Such materials have shown a significant role in synthesizing various biomaterials to serve diverse applications in medical science. The ‘green’, sustainable, biodegradable, and ‘eco-friendly’ polymeric composite materials with renewable and recyclable potential are desired in biomedical applications. Environmental protection is a motivating impulse behind such production of polymeric composites [3]. As recycling approaches are very expensive, the environmental factors related to continuous plastic-based pollution are causing serious global concerns.

Furthermore, the oil resources are limited and costly, thus adding to the recycling problems. Biopolymeric composite materials will potentially resolve the environmental sustainability-pollution conflicts and will decrease fossil fuel dependency [4,5]. Thus, the emphasis was laid on biocompatible and biodegradable polymeric composite materials.

Production of durable, stronger, and lightweight multifunctional biopolymeric materials is desired for biomedical applications. However, developing or choosing approaches to meet the architectural design challenges requires a compromise between visions and objectives, usually conflicting in novel biomaterials [6,7]. It can be further discussing as follows.

(i) Biopolymers: Types of polymers obtained from biological resources, such as living organisms and plants. These are also known as biological macromolecules, biological polymers, or biopolymers.
(ii) Derived biopolymers are polymers that are chemically modified, and their monomers are derived from biopolymers as a starting material like polysaccharides, polypeptides, and other biopolymers.

## 2. Biopolymers

The polymers obtained from living resources or biological origin, including plants, animals, or microorganisms, and their system biology processes are called natural polymers. Carbohydrates, i.e., arabinoxylan, chitosan and starch, proteins, e.g., gelatin and keratin, and polyhydroxyalkanoates (PHAs) such as poly-(3-hydroxybutyrate) [P(3HB)], are all known as biopolymers [8]. Biopolymeric composite materials’ synthesis employs one or more biopolymers to enhance structural and functional properties in the resulting composites [9]. A biopolymer’s composition influences its functional properties, whereas functional potential depends largely on the crystalline or amorphous materials’ behavior. For example, cellulose is a structural polymer where its features are partly due to its crystalline nature.

**Figure 1:**
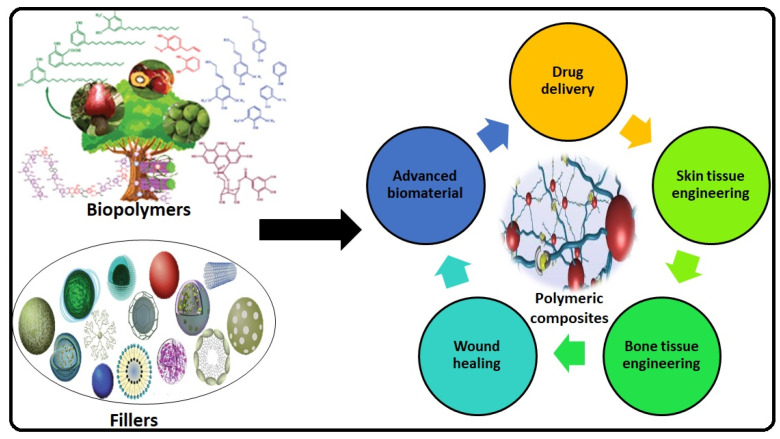
Graphical abstract that describes the different potential application of biopolymers with different fillers.

Nonetheless, physical, chemical, and biological action can turn them into useful multifunctional materials for several purposeful applications [10]. The chemically altered biopolymers, e.g., thiolated arabinoxylan and cellulose acetate, were used in several potential medical and a wide range of applications as shown in the Figure 1. Modified polysaccharides have been extensively used in paint, cement, adhesives, cosmetics, antibacterial coating, medicines, and many other products [11,12].

Chitosan is a well-known compound from the carbohydrate polysaccharide family. It has gained popularity due to bio-sustainability, biodegradability, and compatibility in various fields. It is easily produced from marine sources (lobsters, crabs, shrimp, etc.) and can be used in different biopolymeric composite materials [13]. Among the carbohydrates, arabinoxylan is also a well-known bio-polymers with several potential medicinal applications [14,15]. There are many problems to be considered, such as technical and manufacturing issues before the widespread use of bio-sustainable polymers is possible. An essential obstacle to the broader use of biopolymers in various sectors is their functional characteristics that appear to be unfavorable compared with established petroleum-based polymers. For example, their performance is lower than that of plastic materials since the structural nature of a part of biopolymers with single bonds leads to lower mechanical characteristics [16,17]. These deficiencies can be resolved in various ways, such as grafting, mixing, blending, and reinforcing with other appropriate polymers and ceramics.

### Biopolymer Types

**Figure 2:**
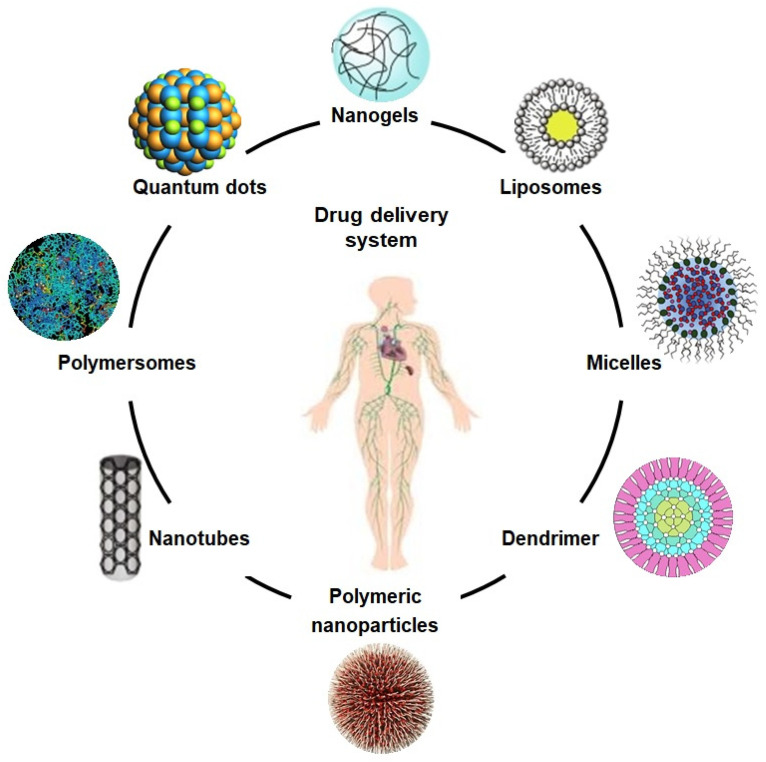
The schematic diagram illustrates different drug carriers for the drug delivery system.

Polymeric composites come from a wide range of sources, from common synthetic materials (petroleum-based) including polystyrene to natural biopolymers like cellulose, proteins, and microbial polyesters, which are important for biological structure and function [18,19]. A broader spectrum of these polymeric materials has been categorized as natural or synthetic according to their source’s nature, as presented in Figure 2. Biopolymers or bio-based polymer composites can be classified into three main categories, depending on their source.

Category 1: Polymers extracted or separated from biomass, such as starch, cellulose, arabinoxylan, and keratin.

Category 2: Polymers obtained from standard chemical processing using renewable biopolymer originated monomers such as polylactic acid (PLA), cellulose acetate (CA), etc., are produced through the fermentation of carbohydrate feedstock.

Category 3: Polymers acquired from microorganisms processing, e.g., mainly PHAs, but bacterial cellulose is under development.

## 3. Bio-Composites

A composite is a generally recognized material composed of one to several different materials or polymers with a marked difference in chemical or physical properties. Composites are used to produce customized properties through enhancing or imparting certain features that cannot be demonstrated by particular homogeneous substances. When polymeric composites contain specific biological phases, they are defined as bio-composites [20,21,22]. Biocomposite materials are synthesized from natural or bio-derived polymers, e.g., chitosan, arabinoxylan, PHAs, or PLA. Efficient and sustainable bio-composites produced from biopolymeric/natural materials and degradable inorganic fillers are called green composite materials. They are the subject of attention because of environmental issues and regulations [23,24]. Bio-based composite materials have currently been produced to target issues in various sectors, like bio-based packaging, biomedical, pharmacy, textile, paper, and others, etc. [25,26]. It started resolving the increasing environmental challenges globally raised by unsustainable non-renewable petroleum resources. Due to growing environmental awareness and regulatory agencies’ requirements, the production and utilization of conventional synthetically manufactured polymers or composite structures are more critically considered [27]. Biopolymers are quite stable materials obtained from various natural resources. They are biodegradable and can be easily recycled, commercially and ecologically viable, and therefore labelled as bio-sustainable products. Environmental factors and microbial decomposition help them degrade to a favorable environment after disposal [25]. The composites of biopolymers, which are biodegradable as well as biocompatible, are termed as “green biocomposites”. Different green biocomposites are reported with numerous inorganic fillers, which includes titania, silica and alumina, etc. [28,29]. Bio-plastic developments delivered a range of feasible and environment friendly products to perform on market already lead by the petroleum based synthetic materials [25].

## 4. Biomedical Applications of Biopolymeric Composite Material

The biopolymers like arabinoxylan, chitosan, guar gum, and xyloglucan have potential benefits over synthetic polymers. These provide a fundamental variety of alternatives in biomedical science for efficient polymeric biomaterials and composite materials. Polysaccharides are potential candidates to give multi-functional, biocompatible, mechanically stable, sustainable, and biodegradable materials. Henceforth, they are emerging as ideal candidates for synthesizing novel composite materials, offering therapeutic efficacy and controlled drug release pertinent for several biomedical applications, and wound remedial and tissue engineering [30]. The polysaccharide-based biopolymeric composites can be employed to fabricate numerous biomaterials like films, fibres, hydrogel, and scaffolds with multifunctional approaches in biomedical engineering.

### 4.1. Drug Delivery

Composites based on polysaccharides, including arabinoxylan, chitosan, and xyloglucan have exhibited an innovative potential as carriers to deliver therapeutic agents, including genes, biomolecules, and biological drugs, as shown in Figure 2. These are used as drug carriers for cancer treatment, cartilage repair, and vascular grafts with excellent biocompatibility, low cytotoxicity, non-antigenicity, process-ability, reversible charging, and releasing mechanisms. Emulsification, gel formation, foaming, and moisture absorption capacities are other assets that enhance their drug delivery application [31,32,33]. Due to their unique mechanical and crosslinking characteristics, desirable biodegradation in different media, and on the targeted sites, these polymeric materials have also been recognized as efficient controlled drug delivery systems [34]. Such biomaterials can be synthesized directly or are incorporated with multifunctional characteristics to engineer specific and targeted sites on resulting nanocarriers. Some reported biomaterials varieties are based on chitosan, guar gum, and arabinoxylan, etc., and have been used efficiently as hydrogels, films, tubes, microspheres, and microneedles [35].

Controlled drug delivery aims to administer therapeutic agents at a steady speed and maintain the effective therapeutic window at the targeted site, usually in blood. It offers economic and desirable therapeutic results that decrease or remove dose-related adverse side effects and toxicity complications and increase patient recovery and comfort. It is believed that the most desirable drug features from biomaterial systems are controlled degradation and sustainable release after accumulation at the targeted site [36]. This controlled release of embedded drugs or any therapeutic agents can be regulated mostly by a trigger such as a temperature, pH, and concentration of ions [30]. In general, the difference between the extracellular matrix’s pH for healthy tissues is 7.4, and in cancer tissues, it is 6.5. Consequently, pH-sensitive polymeric medicines have been efficiently developed for targeted cancer drug delivery [37,38].

The targeted drug delivery system usually needs to activate the cellular sites for a controlled release of therapeutic payloads. Accordingly, a customized drug delivery approach needs to optimize precursors’ synthesis or functionalization, composite manufacturing conditions, and drug encapsulation strategy to fit well with the desired release kinetics [39,40]. Henceforth, all parameters such as size, shape, surface morphology, bioavailability, and biodegradability should be suitable and site-specific to deliver drugs or other nutrients at the targeted site with a controlled rate and dosage [41,42,43,44]. The biomimetic polymeric nanoparticles were synthesized with different sizes and achieved an effective loading of therapeutic agents. Such precise nano drug-carriers helped for molecular imaging of inflamed sites and resolved potential inflammations and immune responses [45,46].

### 4.2. Tissue Engineering

**Figure 3:**
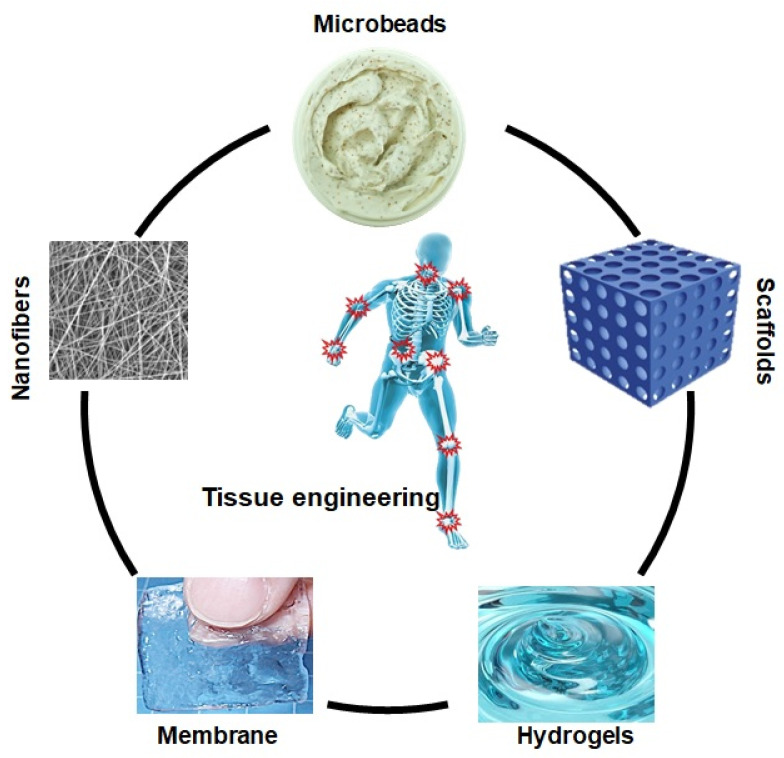
Biopolymer based different form of biomaterials in tissue engineering.

Tissue engineering involves the synthesis of biomaterial scaffolds to treat or regenerate defective tissue, as illustrated in Figure 3. It requires polymeric composite materials with the required composition, desired engineering properties, and adequate physicochemical behavior to support biological tissue growth [47]. After being listed as a subfield of biomaterials, it has grown in depth and significance like an advanced field of its own. Since tissue engineering addresses various applications, the effect is generally associated with applications that replace, repair, or reconstruct part or whole tissue (i.e., bone, cartilage, blood vessels, bladder, skin, muscle, etc.) [48,49]. The tissues concerned often require that certain architectural, morphological, and mechanical properties work properly. Tissue engineering term has also been used to incorporate complex biochemical pathways via cells within the artificially generated protection and support system (e.g., skin, hip replacement, etc.). A schematic diagram has been presented of the role of different biomaterials in tissue engineering in Figure 3.

Tissue engineering was, therefore classified into two types:

Bone tissue engineering.

Skin tissue engineering.

#### 4.2.1. Bone Tissue Engineering

Bone tissue engineering (BTE) is a capable technique that aims at 3D bone scaffolding, containing viable cells and bioactive molecules [50]. BTE focuses on the skeletal structure’s perception, bone dynamics for tissue regeneration as it improves clinical abilities to treat disturbing skeletal and segmental abnormalities [51]. In other cases, the modern science of bone biochemistry and its development is necessary if bone tissue is to be effectively regenerated or restored. A bone may serve a wide multifunctional range that leads to physiological and endocrine stimulation.

Bones constitute the basis of our physical locomotion.

Bones support our load-bearing skeleton and our internal organs with safety.

The bones retain the necessary biological elements for hematopoiesis.

The bones adsorb highly toxic metals due to a porous matrix.

Bones retain vital electrolytes homeostasis by storing calcium and phosphate ions.

The bone undergoes a continuous resorption and reconstruction process that undergoes the exchange of chemicals and structural remodelling due to internal intermediaries and external mechanical standards. Bone was historically and most accurately named the ultimate intelligent material because of its scarce regenerative adaptability. Functional bone tissue engineering includes the fusion of the newly healed bone with the adjacent host bone and, most significantly, the native bone functions [52,53,54]. The bone is an extremely complex tissue due to functional and architectural diversity. The bone extracellular matrix (ECM) particularly consists of both an organic non-mineralized matrix and an inorganic mineralized component. The nanocomposite structure is important for the compressive strength needed and thigh bone fracture resistance and load-bearing applications [55,56]. In either case, cellular mesenchymal condensation occurs firstly that acts mostly as a platform for osteogenesis, and mesenchymal progenitor cells, extracellular matrix development is necessary. These cells differentiate in osteoblasts and ultimately build portions of the mandible, clavicle, and several other cranial bones. Furthermore, endochondral bone formation forms a substantial portion of bones. This technique includes the first differentiation of mesenchymal progenitors into the chondrocytes responsible for the deposition of a cartilage template that is later mineralized and replaced by bone [57,58,59].

Various engineering methodologies could include adequate extracellular matrix molecules or adherent ligands that stimulate stem cells and mediate earlier to regenerate bone tissues. Bone tissue engineering should be aimed at fabricating scaffolds to promote angiogenesis that incorporates growth factors and has the porous structure needed for vascular growth [60,61]. Tissue engineering of these scaffolds with micro and nano-meter surface morphology is essential for cellular adhesion, proliferation, and differentiation [62,63]. More broadly, the development of polymeric scaffolds can enhance osteogenesis that encourages the remodelling of tissues. Effective bone regeneration needs to support the function of bone tissue.

Subsequently, bone tissue therapies in hospitals need a more definitive strategy, such as using in-vitro scaffold regeneration machines to restore bone functions in vivo. 3D printing or additive manufacturing (AM) has been frequently used in different industries, including construction, design and development, biomechanical, and tissue engineering [64,65]. The key advantage of 3D printing is the opportunity to produce complicated shapes, as it can produce parts of different sizes from micro to macro and allow fast prototyping. Producing products via 3D printing has reduced the additional costs, and personalized products can be printed in small quantities [66]. The most common materials involved in 3D printing are polymers, metals, ceramics, and concrete [65].

Nonetheless, poly(lactic) acid (PLA) is among the synthetic polymers used primarily for 3D scaffold printing [67]. It is necessary to choose the right biopolymer to have the desired tissue engineering features, and the scaffold may work properly after implantation [61]. Some ideal composite biomaterials based on biopolymers have been reported as a better option for the potential tissue engineering system (Table 1).

#### 4.2.2. Skin Tissue Engineering

The skin is considered one of the most sensitive organs due to its multi-functional roles, such as acting as a protecting enclosure for internal organs’ safety, regulating the body’s temperature, and acting as a sense organ [93,94]. Normal skin consists of three components known as epidermis, dermis, and hypodermis.

The outmost waterproof layer is the epidermis, which acts as a defensive barricade against pathogens and foreign materials, and plays a critical role in body temperature and humidity regulation [95,96]. Keratinocytes make up more than 90% of most cells of the epidermis. The majority of the epidermal cell population is dominated by Langerhans, Melanocytes, and Merkel cells [97]. Dermis makes up about 90% of the skin’s weight and forms the skin’s base [95,98]. It is a soft tissue composed of extracellular matrix (ECM), many types of cells, glands, and hair follicles. The dermis layer is well-vascularized by blood vessels and contains nerve endings. Fibroblasts are the largest dermal cells, which contain collagen and elastin, and provide mechanical strength and elasticity to the skin [99,100]. The hypodermis is a deeper level of elastic fibrous tissue with mucous properties, skin cells that store fat, blood vessels, and nerves [101,102]. Traumas such as physical piercing, poisoning, fire, disease, or surgery damage this main organ and lead to the vital organs at risk of infection, injury, or dehydration. Skin replacement tissue engineering provides a potential basis for better care in the fight against chronic and acute skin wounds [103,104]. However, as the mechanical and physiological parameters of active skin concerns, the skin tissue engineering necessitates having a cell base and simulated extracellular matrix (ECM) to communicate with the surrounding tissue [105]. There are currently no major skin prototypes that accurately reproduce the structure, composition, organic consistency, or visual atmosphere of natural skin. Skin alternatives may have important features as easy to use and specific to the type or the position of the wound. These biomaterials have adequate aquatic fluidity and special adherents towards host sites [106,107]. They have sufficient biochemical and mechanical properties, have controlled deprivation, are disinfected, non-toxic and non-antigenic, and have negligible inflammatory effects. They can also join the congregation with minimal harm and angiogenesis pain while still at operating costs. The ultimate goal of tissue engineering is to attain the maximum of these requirements to prepare smart skin replacements [97,108,109,110]. Earlier skin replacements faced failure, contamination, and defects, which led to the restricted use of autographs and allografts. Recently, synthesized skin substitutes have developed with the multifunctional properties. Henceforth, the extension of an imitated skin provides relief for patients with burns and other skin-related disorders [111,112,113]. These studies provide a summary of the improvements and potential alternative materials for skin care and treatment to regenerate targeted tissues.

Autologous keratinocytes are formed and grown in interconnected layers of the epithelium, and these are substituted for those with large skin deficiencies. Approximately, after more than 140 replications, we can isolate clonogenic keratinocytes or holoclones from the skin and gradually increase culture. In many studies related to tissue engineering, polymeric materials have multifunctional properties to support stem-cell growth and differentiation. These studies have been proved multifunctional properties of polymeric material to grow stem cells based on their abilities to regenerate various skin lines [114,115]. The stem cells embedded in epidermal layers promote the repair and regeneration of the epidermis. The growth of epidermal stem cells in allogeneic dermis or fibrin has proved suitable for quick growth and regeneration processes [116]. Growing epidermal stem cells as autologous sheets to cover the major epithelial area, in cases such as burns, take only more than a few weeks [103,117]. In this way, mounting stem cells on a polymeric material from a minor skin biopsy reduces the time required to label outsized epithelial layers. Besides, fibrin or allogeneic dermis epidermal stem cells maintain their wound healing ability [118,119]. Somehow, such epidermal stem cell implants lack adequate skin regeneration. Efforts to integrate the sophisticated epidermal parts such as hair follicles, sweat glands, and sebaceous glands could not meet success, which indicates that multifaceted connexions of epithelial and mesenchymal layers are important for the generation of additions [120,121]. Furthermore, the polymeric composite materials do not reinstate the novel skin’s electronic properties or visual structure. Changes in stem cell biology and skin morphogenesis are necessary for extending skin developments, delivering the usual usefulness and aesthetics of healthy skin [122].

### 4.3. Wounds Healing

Wounds are an irregular skin puncture, breakage, or deformity of the skin due to thermal/physical damage or chronic disease [123]. Depending on the healing technique, the wounds may be listed as chronic or acute wounds. Chronic wounds are mainly tissue lesions that appear to settle completely, typically within 8 to 12 weeks. Acute injuries continue to reappear and still have a recovery time of more than 12 weeks [124,125,126]. Different neural aspects can lead to impaired wound healing of a wound or an inability to heal properly. Examples of chronic wounds include bedsores (ischemic or venous) and leg ulcers [127,128]. Skin layers and contaminated sites are used as a basis for wound gradation, and surface wounds involve only the epidermal skin surface. The word “partial-thickness wound” refers to injuries affecting the epidermis, deep epidermis layer, muscles, soft tissue, and follicles. Besides the epidermis and the dermal surface, wounds of maximum thickness are associated with subcutaneous fat or deep tissue [129,130,131].

The physiological regeneration of wounds includes coordinated cooperation between different biological systems. It comprises a cascade of regulated activities to heal a wound completely. Hysteresis and blood clotting begins as a lesion, occurs in any part of the body, and the main objective of such processes is to prevent instant exsanguinations. This lesion is also a secondary long-term target and a matrix for adhesion to invading cells [132,133]. Homeostasis and the quantity of fibrin produced at the injury site are dependent on a properly regulated balance of the endothelial cells, thrombocytes, coagulation, and fibrinolysis. Vascularization is caused by the neurological reflex system in impaired vessels, thereby blocking blood flow for several minutes. Homeostatic behaviors and proliferation and differentiation cause the waterfall of coagulation [134,135]. Platelets bind to the extracellular matrix when blood spills, inducing the clotting factor’s release: fibronectin, fibrin, vitronectin, and thrombospondin. Clotting retains homeostasis and a matrix for migrating cells in corresponding to homeostatic and inflammatory procedures [136,137,138].

Many biopolymers, including fibrous proteins and various polysaccharides, are commonly engaged in wound care and treatment. These biocompatible, biodegradable polymeric matrixes maintain an atmosphere analogous to the extracellular environment and speed up the usually slow wound healing process [15,139,140]. The biopolymeric matrix offers an excellent micro-environment for cell adhesion, proliferation, cell migration, and differentiation. Three-dimensional crosslinked polymeric networks can maintain enough moisture and oxygen-based on wound treatment materials made from biopolymers at the wound site. The wound healing dressings promote regeneration, prevention, and protection of the wound, especially from severe disease-causing pathogens. It is important for repairing and regenerating the dermal and epidermal tissues [141]. These wound healing materials are recognized as hydrogels that can be packed with spatially and temporally regulated cells, medicines, and peptides for localized therapeutic delivery [142,143]. Biopolymeric based potential biomaterial has been summarized in Table 2.

Hydrogels were utilized in biomedical research and clinical applications, including tissue engineering, regenerative medicine, cancer treatment, infectious diseases, controlled drug delivery, and peptide delivery [183,184]. The loading and subsequent release of bioactive molecules into hydrogels can be managed by regulating the crosslinking rate, which results in many pharmacokinetics control options for developing optimum hydrogel-releasing therapeutics agents or bioactive molecules [185,186,187,188]. Hydrogels can progressively elucidate chemotherapeutic drugs in local settings, which can help to discharge therapeutic agents systemically. The high moisture content of hydrogels and the physicochemical and biological similarities with the native extracellular atmosphere make them principally biocompatible and practicable for substantial therapeutic applications [189,190]. Hydrogels conform to the shape of the application site, making the formulation of loaded hydrogels much more clinically feasible in biomedical applications. While poly (ethylene glycol) (PEG) use in hydrogels was considered to be extremely biocompatible, e.g., PEG hydrogels, PEG-based hydrogels improve fibroblast growth rates [191,192]. PEG’s high systemic biocompatibility and use of ECM-derived biomaterials improve cellular growth distribution. As a result, PEG-based crosslinked hydrogels evolve as multifunctional materials for wound care technology with favorable loaded materials like cells, drugs, and peptides [193,194,195].

## 5. Advanced Functional Biomaterials

A better understanding of the sequential, structural, and functional characteristics of natural polymers plays a substantial role in designing and synthesizing multifunctional polymeric materials. These advanced artificial biomaterials have abilities of self-assembly and stimuli response under certain conditions to promote cell interaction and proliferation [196,197,198]. The complexity of post-transcriptional alterations restricted the synthesis of advanced and multifunctional protein biomaterials utilizing bacterial resources, and the paradox of target genes being inserted into the sequences. Other modifications were designed to control spatial and temporal releases correctly. Advances in gene therapy and DNA manipulation methods have enabled structural, and de novo design developments for protein-based biomaterials [199,200,201]. These allowed the development of polymer composites of nano-size with considerable physicochemical and biochemical properties. These characteristics can be linked to essential design modules from a modular field of composite polymeric materials such as collagen, elastin, silk, and resilin [202,203,204]. The structure of such synthesized biomaterials is associated with great versatility, such as cell-binding sites and enzymatic domains, due to the presence of multi-functional domains on protein structure.

Recent developments in genetic engineering have promised the design and synthesis of advanced biomaterials based on artificial proteins. These biomaterials have unique performance compared to their native counterparts, such as enhancing the self-assembly into the fibrous structures [205,206]. Different cloning pathways, such as yeast, bacteria, plant, and mammalian cells have already been studied to express native and synthetic protein-based biopolymers [141]. These biopolymers imitate proteins’ primary modular structure having distinct physicochemical and biological characterizations [207,208,209]. Escherichia coli (E. coli) is widely used to manufacture protein biopolymers such as silk, elastin, resilience, and other biomimetic proteins [144].

## 6. Conclusions and Future Directions

This review article aims to address the possible biopolymers, biocomposite materials, and potential applications in drug release, tissue engineering, wound healing, and advanced functional biomaterial. Previously, petroleum-based synthetic polymers were considered the best biomaterials in tissue engineering and regenerative medicines. Still, due to depletion in resources, biodegradability, eco-friendly and environmental sustainability make scientists and research to replace them with other biopolymers. The biopolymers are the best substitutes for synthetic polymers obtained from petroleum with considerable renewable, biodegradable, environmental, and eco-friendly properties. The biopolymers don’t support mechanical properties like high tensile strength, impact strength, flexural strength, thermal stability, etc. However, their ceramic composites display sufficient mechanical strength to support load-bearing applications. Still, further attention, developments, and improvements are necessary. The traditional blending techniques have been adopted to tailor the microstructural properties of biocomposite by adding reinforcements. Sometimes, their structural properties are also enhanced by blending them with synthetic polymers (PVA, PMMA, etc.). Different fillers have been recently introduced into the biopolymers to prepare highly functional composites for the biomedical industry.

Study in this field has grown dramatically in recent decades, which has been confirmed by the resulting rise in the number of newly published materials. In many industries, including packaging, vehicles, building, electronics, and most significantly, biopolymer composites find countless daily applications in medicine. The widely used biochemical recipes for biopolymer composites synthesis include injecting, extrusion, and in situ synthesis. The introduction of graphene and its derivatives as fillers into biopolymers strengthened their mechanical properties to obtain desired biomaterials, such as tensile, effect, flexural, and other structural properties. The metallic nanoparticles also have played a vital role as fillers to synthesize biocomposite to enable several opportunities to explore their innovative properties in medical applications. Mechanical behavior and poor dispersion are the major problems that limit the use of biopolymers to synthesize biocomposites. The fillers form agglomerates with a biopolymer matrix, resulting in poor interfacial bonding resulting in poor mechanical properties with poor structural uniformity. Such composites result in many other odd parameters, including high-temperature sensitivity, moisture sensitivity, low impact strength, and shelf life, etc. Future directions lead towards new biomaterial to address the stated factors and fit well with the economic viability, recycling processes and being eco-friendly.

## Figures and Tables

**Table 1:** Polymeric composite materials in tissue engineering.

Sr. No	Polymers	Salient Features	References
1.	Chitosan	Biodegradable, biocompatible, antibacterial, cytocompatible, bioactive	[68,69,70,71]
2.	Gelatin	Bioactive, biocompatible, hemocompatible, cell adherence, anti-thrombogenic	[72,73,74,75]
3.	Arabinoxylan	Biocompatible, antibacterial, cell adherence, bioactive, cell proliferation	[14,76,77]
4.	Collagen	Biodegradable, fibrous, biodegradable, cell proliferation	[70,78,79,80]
5.	Xyloglucan	Cell proliferation, biodegradable, cell differentiation, biocompatible	[81,82]
6.	Fibrinogen	Biocompatible, hemocompatibility, cell proliferation, biodegradable, cytocompatible	[83,84,85,86]
7.	Hyaluronic acid	Bioactive, cell adherence, cell proliferation, cell differentiation, biocompatible	[87,88,89,90]
8.	Beta-glucan	Biocompatible, bioactive, biodegradable, antibacterial, mechanical	[91,92,93]

**Table 2:** Biopolymeric materials as a potential biomaterial in wound healing.

Sr. No	Polymers	Salient Features	References
1.	Arabinoxylan/guar gum/gelatin/collagen	antibacterial, biocompatible, biodegradable, bioactive, sustained drug release, cell proliferation	[15,144,145,146,147]
2.	Beta-glucan/chitosan	biocompatible, antibacterial, cell proliferation, bioactive, bioactive molecule release, adherence	[148,149,150,151,152,153,154]
3.	Alginate/Fibrinogen/Hyaluronic acid/xyloglucan	Fibrous protein, biocompatible, biodegradable, fibrous, antibacterial, cell adherence, cell proliferation	[155,156,157,158,159,160,161,162,163]
4.	Bacterial cellulose/pectin/carrageenan	antibacterial, cell adherence, cell differentiation, biocompatible, bioactive, cytocompatible	[164,165,166,167,168,169]
5.	Fucoidan/Silk sericin/keratin	anti-coagulant, anti-inflammatory, anti-viral, anti-tumor, anti-thrombic	[170,171,172,173,174,175,176]
6.	Bovine serum/agar/Acetobacter xylinum	cell proliferation, cell adherence, biocompatible, bioactive, antibacterial, cytocompatible	[164,177,178,179,180,181,182]

## Data Availability

Data presented in this study are openly available and cited in the references.
